# A Comparative Study on Two Cationic Porphycenes: Photophysical and Antimicrobial Photoinactivation Evaluation

**DOI:** 10.3390/ijms161125999

**Published:** 2015-11-12

**Authors:** Rubén Ruiz-González, Montserrat Agut, Elena Reddi, Santi Nonell

**Affiliations:** 1 Institut Químic de Sarrià, Universitat Ramon Llull, via Augusta 390, I-08017 Barcelona, Spain; montserrat.agut@iqs.url.edu; 2 Department of Biology, University of Padova, via U. Bassi 58/B, E-35121 Padova, Italy; elena.reddi@unipd.it

**Keywords:** photodynamic therapy, antimicrobial photoinactivation, MRSA, *P.aeruginosa*, photosensitizer, porphycene, flow cytometry, singlet oxygen

## Abstract

Over the last decades, the number of pathogenic multi-resistant microorganisms has grown dramatically, which has stimulated the search for novel strategies to combat antimicrobial resistance. Antimicrobial photodynamic therapy (aPDT) is one of the promising alternatives to conventional treatments based on antibiotics. Here, we present a comparative study of two aryl tricationic porphycenes where photoinactivation efficiency against model pathogenic microorganisms is correlated to the photophysical behavior of the porphycene derivatives. Moreover, the extent of photosensitizer cell binding to bacteria has been assessed by flow cytometry in experiments with, or without, removing the unbound porphycene from the incubation medium. Results show that the peripheral substituent change do not significantly affect the overall behavior for both tricationic compounds neither in terms of photokilling efficiency, nor in terms of binding.

## 1. Introduction

There are approximately 1400 known species of human pathogens, but they account for much less than 1% of the total number of microbial species on the planet [1]. Despite this fact, they have arisen as one of the main emerging threats worldwide. The use and abuse of antibiotics has enabled the emergence of the so-called *superbugs* or ESKAPE pathogens (*Enterococcus faecium*, *Staphylococcus aureus*, *Klebsiella pneumoniae*, *Acinetobacter baumannii*, *Pseudomonas aeruginosa*, and *Enterobacter* spp.) [2]. These superbugs, extremely difficult and in many cases impossible to effectively treat, have situated infectious diseases as the second most important killer in the world [3]. In the antibiotic era, efforts were focused on discovering novel natural antibiotics, developing semi-synthetic antimicrobial agents or modifying currently known active molecules. However, therapeutic success of classical treatments is declining and alternative approaches have been suggested [4,5,6,7,8]. Some of these alternatives are not novel, but their use was eclipsed at the time of their discovery mainly due to insufficient understanding of their basis and the advent of antibiotics.

Photodynamic therapy (PDT) combines light, oxygen, and a photoactive drug (photosensitizer, PS) to generate reactive oxygen species (ROS) capable of exerting localized oxidative damage. Among these ROS, singlet oxygen (^1^O_2_), the lowest electronically-excited state of molecular oxygen, is endowed with rather unique properties, especially relevant for application in biological systems: it is small and, therefore, capable of diffusing with relative ease; it is non-charged, which allows it to cross membranes; it is fairly reactive, and there are no known antioxidant enzymes for removing it [9,10]. All these attributes have proven PDT useful in different fields, especially in the battle against cancer [9,11,12].

The increasing understanding of the factors affecting the efficiency of antimicrobial photodynamic therapy (hereafter aPDT), together with new developments in the field, suggest that aPDT may be a promising approach for the treatment of superficial and localized infectious diseases [13,14]. Despite promising *in vitro* results, aPDT is not being routinely applied in clinical settings yet. However, clinical studies have been carried out especially in dentistry and dermatology [15,16,17]. Advantages of aPDT over traditional antimicrobials include broad-spectrum activity (aPDT is also active against virus, fungi, protozoa, and bacteria, including antibiotic-resistant strains) and lack of development of resistance mechanisms due to its multi-target mode of action [18]. One extra attractive feature is the possibility that the generated ROS may chemically destroy many of the secreted virulence factors [18]. Moreover, aPDT has become a powerful research tool: to help identify the photophysical mechanisms involved in light-mediated cell inactivation in order to develop potent and clinically-compatible PSs; to understand how photoinactivation is affected by key microbial phenotypic elements (multidrug resistance and efflux, virulence, and pathogenesis determinants, growing in biofilms); to explore novel delivery platforms inspired by current trends in pharmacology and nanotechnology and to identify photoinactivation applications beyond the clinical setting, such as environmental disinfectants [3].

**Scheme 1:**
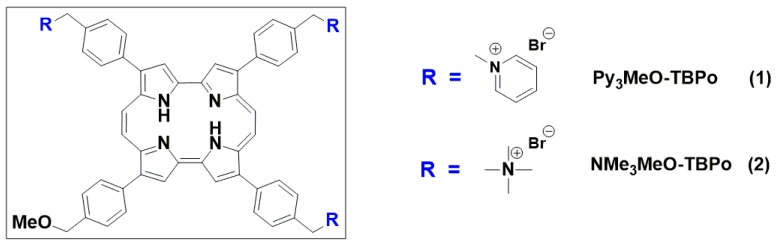
Aryl tricationic porphycenes under study.

Unlike neutral and anionic PSs, cationic PSs at physiological pH are capable of accomplishing efficient photoinactivation in both Gram-positive and Gram-negative bacteria without the need for any co-administered agent [19,20,21,22,23,24,25]. The present work is a comparative study of two tricationic PSs of the porphycene family (Scheme 1) as potential third-generation PSs. Porphycenes have long been regarded as an interesting family of PSs because of their appealing optical and photochemical properties conferred by their lower molecular symmetry in comparison to porphyrins [26].

A novel tricationic porphycene, namely, 2,7,12-tris(trimethyl-*p*-tolyl)–17-(*p*-(methoxymethyl)phenyl) porphycene (NMe_3_MeO-TBPo, **2**) was synthesized and compared against its predecessor, 2,7,12-tris(α-pyridinio-*p*-tolyl)–17-(*p*-(methoxymethyl)phenyl) porphycene (Py_3_MeO-TBPo, **1**) [27]. There is ample evidence in the field of antimicrobial photodynamic therapy that significant differences exist between the antimicrobial activity of trialkylammonium- *versus* pyridinium-substituted photosensitisers. For instance, Falk *et al.* reported that a trimethyl-anilinium derivative of hypericin displayed a pronounced photodynamic inactivation of the *Propionibacterium acnes* after illumination, whereas the photobactericidal efficacy of the *N*-methyl-pyridinium derivative was negligible under identical experimental conditions [28]. Totally different observations were made using pyridinium- and anilinium-substituted porphyrins depending on the bacteria studied. Thus, for *Enterococcus seriolicidia* (Gram-positive) inactivation was higher after photosensitization with meso-tetra(4-*N*,*N*,*N*-trimethyl-anilinium)porphyrin as compared to meso-tetra(4-*N*-methyl-pyridynium)porphyrin, whereas for *Vibrio anguillarum* (Gram-negative) the pyridinium analog was more efficient in cell killing [20]. More recently, a series of trialkylammonium and pyridinium derivatives of the photosensitiser phenalenone were compared and, again, important photodynamic activity differences were observed among them [29]. We have, therefore, comparatively analyzed the photoinactivation ability of both cationic porphycenes against model Gram-positive and Gram-negative bacteria and yeasts and relate it to their photophysical properties by a wide range of spectroscopic techniques and to their binding affinity to bacteria.

## 2. Results and Discussion

### 2.1. Photophysical Characterization of Porphycene Derivatives

#### 2.1.1. Absorption and Fluorescence

**Figure 1:**
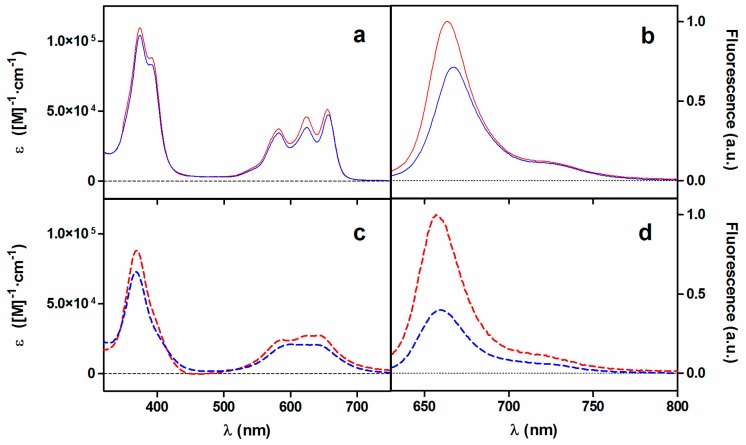
Absorption (**a**,**c**) and normalized fluorescence (**b**,**d**) spectra of **1** (red) and **2** (blue) in MeOH (**a**,**b**; solid line) and water (**c**,**d**; dashed line), respectively.

The main photophysical properties of porphycene **2** are summarized in Table 1 and compared to those already reported for the tricationic analogue **1** and the non-cationic parent 2,7,12,17-tetraphenylporphycene (TPPo) [27,30]. Panels a,c in Figure 1 show the absorption spectra of both tricationic porphycenes in methanol (MeOH) and water, respectively. The substituent change does not result in remarkable spectral profile differences for both compounds except for a 2-nm redshift for **2**. The porphycene’s characteristic main band in the Soret region plus a small shoulder and three bands in the Q-region can be distinguished in MeOH while a remarkable loss of structure occurs in aqueous media due to aggregation. Furthermore, the absorption coefficient values (ε) are smaller in both media for compound **2**.

**Table 1:** Summary of photochemical properties of the porphycenes under study.

Compound	Solvent	λ_Abs_/nm ^a^	λ_Fluo_/nm ^b^	Φ_F_^c^	τ_S_/ns	Φ_Δ_^d^	τ_Δ_/μs
**TPPo ^e^**	Toluene	659 (5.0 × 10^4^)	667	0.150	4.8	0.230	-
**Py_3_MeO-TBPo (1)**	MeOH	655 (5.1 × 10^4^)	664	0.075	2.6	0.193	10.0
	Water	644 (2.6 × 10^4^)	656	0.005	1.8	0.004	3.7
**NMe_3_MeO-TBPo (2)**	MeOH	657 (4.7 × 10^4^)	669	0.054	2.6	0.180	9.6
	Water	641 (2.0 × 10^4^)	660	0.002	2.0	0.003	4.1

**^a^** Maximum of the lowest energy absorption band with ε values (M^−1^·cm^−1^) in brackets; **^b^** Maximum of the fluorescence band; **^c,d^** Values at 532 nm excitation wavelength; **^e^** Data from reference [30].

Regarding the shape of the fluorescence spectra (panels b,d in Figure 1), no significant differences can be observed between both compounds other than minor spectral shifts. The spectra show the typical features of other porphycenes, namely a main band with a weaker vibrational overtone at lower energy that are mirror images of the S_1_←S_0_ absorption transitions [26]. The fluorescence excitation spectra in water (Figure S1) show the structure of the absorption spectra in MeOH, confirming that non-fluorescent aggregates are formed. The fluorescence quantum yield values (Φ_F_) for **2** in MeOH and water were lower than those for **1** as deduced from the ratio of intensities (1.4- and 2.5-fold, respectively; see panels b,d in Figure 1 and Table 1).

As for the singlet state kinetics, fluorescence decays could be fitted to a monoexponential decay function (see Figure S2), consistent with the previous observation that only the monomeric species are fluorescent [27]. The fluorescence lifetime values (τ_s_) are equal in MeOH for both compounds while in aqueous media the lifetime value is slightly longer for **2** (Table 1).

#### 2.1.2. Near-Infrared Phosphorescence

**Figure 2:**
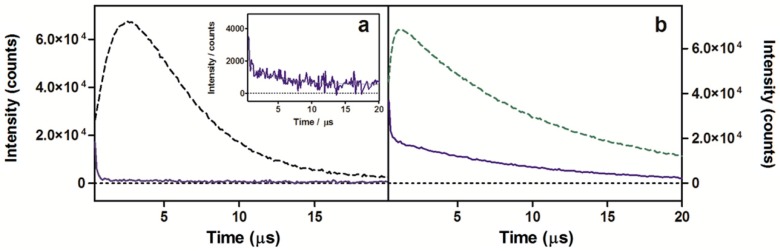
^1^O_2_ phosphorescence signals observed at 1275 nm for optically matched solutions at 532 nm of porphycene **2** (blue solid lines) and reference PSs (green dashed lines) in water (**a**) or in MeOH (**b**). Inset: signal magnification of **2** in water. 5,10,15,20-tetrakis(*m*-hydroxyphenyl)-21H,23H-porphine (*m*-THPP) in MeOH (**a**) and 5,10,15,20-tetrakis(*N*-methyl-4-pyridyl)-21H,23H-porphine (TMPyP) in water (**b**) are the chosen reference PSs.

^1^O_2_ production was detected through direct observation of the ^1^O_2_ phosphorescence maximum centered at 1275 nm [31] for samples excited at 532 nm (Figure 2). Analysis of the signals for compound **2** resulted in decay lifetimes that matched the ^1^O_2_ lifetime values in neat water and MeOH found in the literature (3.4 and 9.8 μs, respectively) [32].

In order to get an estimation of the photosensitizing capacity of the new tricationic compound, the ^1^O_2_ quantum yield (Φ_Δ_) was calculated. To this aim, optically-matched solutions of a reference PSs (TMPyP; Φ_Δ_ = 0.74 in water; *m*-THPP; Φ_Δ_ = 0.69 in MeOH) [33,34] and porphycene **2** were measured, the signals were adjusted to monoexponential decay functions and the intensity of the ^1^O_2_ lifetime value (τ_Δ_) were compared (Figure 2 and Equations (2)–(4) in Experimental Setion). Table 1 summarizes these results and evidences lower capacity of generating ^1^O_2_ for the new tricationic derivative both in water and MeOH.

### 2.2. Photodynamic Inactivation Studies

**Figure 3:**
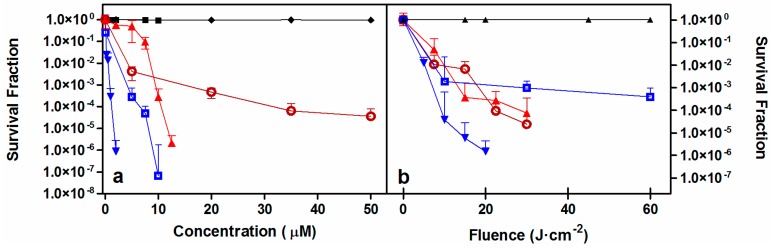
Photoinactivation studies against *S. aureus* (filled blue inverted triangles) *E. coli* (open blue squares), *C. krusei* (filled red triangles) and *C. albicans* (open red circles). Concentration-dependent (**a**) and light-dependent (**b**) survival curves for compound **2**. Controls: No light (black diamonds). No PS (black triangles). Solid lines connecting data series are provided only for visualization purposes. Fluences: 20 J·cm^−2^ for *S. aureus*, 60 J·cm^−2^ for *E. coli* and 22.5 J·cm^−2^ for both *Candida* species, respectively. PS concentrations: 2 μM against *S. aureus*; 5 μM against *E. coli;* 10 μM against *C. krusei*, and 35 μM against *C. albicans*.

As a first approach, porphycene **2** was tested against representative members of the Gram-positive and Gram-negative families (namely *Staphylococcus aureus* and *Escherichia coli*) as well as two species of yeast (*Candida albicans* and *C.* *krusei*). Reductions of bacteria population of up to 6-log_10_ in colony forming units per millilitre (CFU/mL) and at least of 4-log_10_ for *Candida* species could be achieved in a light-dose and PS-concentration dependent fashion (Figure 3). Results were comparable to those previously reported for **1**, albeit **2** was slightly less efficient. Dark controls revealed less than 1-log_10_ reduction in CFU/mL at all the assayed concentrations.

**Figure 4:**
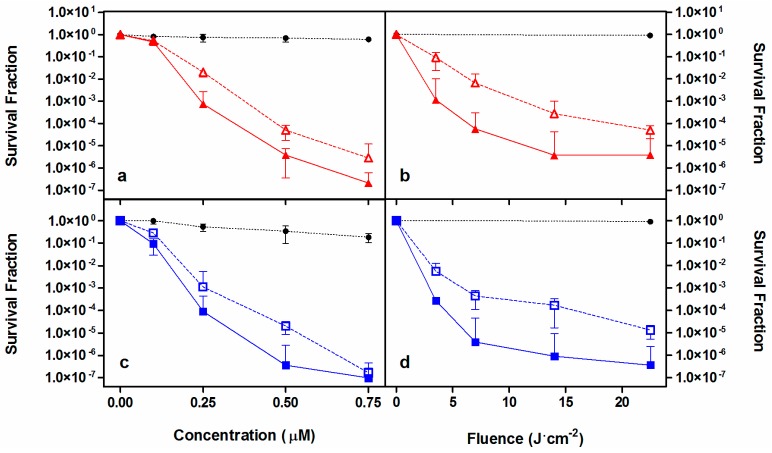
Photoinactivation studies against MRSA. Survival curves of **1** (red, **a**,**b**) and **2** (blue, **c**,**d**) removing (empty symbol) or not (filled symbol) the PS excess from the solution before irradiation. Solid and dashed lines connecting data series provided for visualization purposes. Black symbols are the controls. Fluence was maintained at 22.5 J·cm^−2^ (**a**,**c**) and PS concentration of 500 nM was assayed (**b**,**d**).

Once its capacity as photosensitizing agent was corroborated, **2** was challenged against two members of the ESKAPE family pathogens, namely methicillin-resistant *S. aureus* (MRSA) and *Pseudomonas aeruginosa* and directly compared to **1**. A reduction over 6-log_10_ in CFU/mL was achieved against MRSA with both porphycenes at concentrations below 750 nM at a constant fluence of 22.5 J·cm^−2^ (Figure 4, panels a, c). When concentration was held at 500 nM, a population reduction up to 6-log_10_ CFU/mL could be achieved by increasing the fluence to approximately 20 J·cm^−2^ (Figure 4, panels b, d). After the samples were centrifuged to remove excess unbound PS and bacteria were resuspended in sterile PBS prior to irradiation, a small decrease in the photokilling efficiency of approx. 1-log_10_ was found. This trend could be observed for both PSs under almost all conditions assayed.

**Figure 5:**
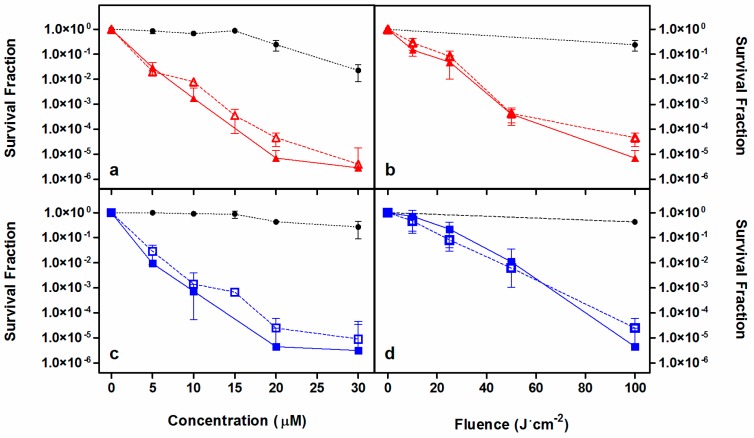
Photoinactivation studies against *P. aeruginosa*. Porphycenes **1** (red, **a**,**b**) and **2** (blue, **c**,**d**) survival curves removing (empty symbol) or not (filled symbol) the PS excess from the solution before irradiation. Black symbols are the controls. Solid and dashed lines connecting data series are provided for visualization purposes. Fluence was maintained at 100 J·cm^−2^ (**a**,**c**) and PS concentration of 20 μM was assayed (**b**,**d**).

Analogue studies were carried out with the hard-to-treat Gram-negative *P. aeruginosa.* As shown in Figure 5, an approximately 6-log_10_ reduction was achieved with both PSs in the two series of experiments, although higher doses of either light or drug were necessary in comparison to *S. aureus* and *E. coli* analog experiments. Two relevant observations can be drawn out of these experiments. First, PS **1** shows higher dark toxicity than PS **2** at concentrations higher than 20 μM. Second, the differences in photoinactivation with, or without, removing the excess of PS lie within experimental error, suggesting strong binding of these cationic compounds to the cell wall of these Gram-negative bacteria.

### 2.3. Flow Cytometry Analysis

Photoinactivation studies suggested that cell-death was mainly due to bound PS molecules, particularly in *P. aeruginosa*, as deduced from the minor effect of washing out the unbound PS. Flow cytometry experiments were carried out in order to evaluate the interaction of the PSs with MRSA and *P. aeruginosa* cells under the same conditions as in the photoinactivation studies.

**Figure 6:**
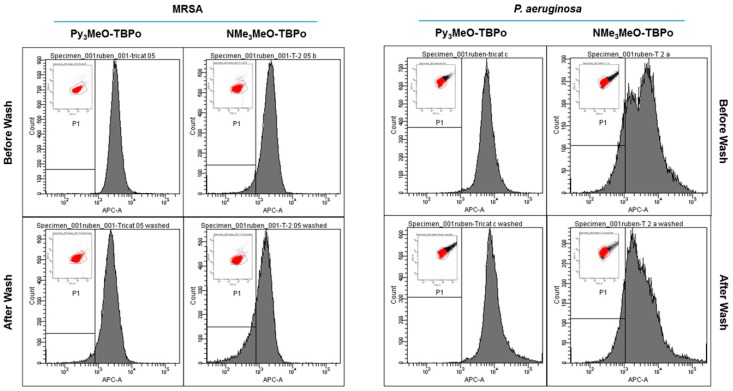
Flow cytometry analysis of bacterial cultures incubated with either **1** or **2** against MRSA (**left panels**) or *P. aeruginosa* (**right panels**). Cell fluorescence measured in the optimum conditions with or without removing the PS excess. Inset: density plots and ROI of the gated bacteria.

Flow cytometry analysis was carried out at the optimum aPDI conditions, namely 30 min incubation in the dark with either 500 nM and 20 μM porphycene for MRSA and *P. aeruginosa*, respectively. Incubated samples were analyzed with or without removing the PS excess. Figure 6 shows the histograms of the PS fluorescence distributions in MRSA (left panels) and *P. aeruginosa* (right panels) and supporting Table S1 gathers the mean fluorescence of the selected populations.

The flow cytometry histogram for MRSA shows a slight shift of the fluorescence maximum to lower fluorescence intensities but the mean fluorescence value does not show any detectable decrease (Table S1). These results indicate that removal of the PS after a washing step is not significant for any of the tricationic porphycenes. Analog results for *P. aeruginosa* showed that the differences in cell fluorescence with or without removing the excess PS lie within experimental error (*p* = 0.33). These observations very much correlate with the photoinactivation outcome presented in the previous section. Interestingly, the histograms were not as narrow in comparison to those for MRSA, especially in the case of porphycene **2** where two cell populations with different fluorescence intensities could be distinguished.

### 2.4. Discussion

Porphycenes are structural isomers of porphyrins and constitute an interesting family among 2nd generation PSs. Porphycene derivatives show higher absorption values than porphyrins in the red spectral region due to their lower molecular symmetry [26]. Their photophysical and photobiological properties make porphycenes excellent candidates for photodynamic applications and, in fact, they have proven successful in recent studies addressing not only antimicrobial targets but also tumor diseases [27,35,36,37]. Based on the encouraging results obtained with the first tricationic aryl porphycene **1** against a wide variety of pathogenic species both *in vivo* and in animal models [27], a second member of the family of the tricationic porphycenes was synthesized. Decades of research on porphycenes has built the knowledge that most dramatic photophysical changes arise either by increasing the conjugation (electronic circulation within the macrocycle) or by introducing heteroatoms to the porphycene core [26]. Thus, one would not expect remarkable changes due to substituent changes in the peripheral tolyl groups. Notwithstanding spectral changes were minor, a general decrease in the photophysical parameters for PS **2** was found (Table 1). For instance, a reduction in the Φ_F_ value was observed, not fully in line with the decrease in the τ_s_ value. Still, both porphycene’s photophysics are similar and lie far from those of the parent non-cationic TPPo. For instance, the τ_s_ values are almost half faster as compared to TPPo in toluene (4.8 ns), which can be attributed both to new deactivation pathways, due not only to intermolecular interactions consequence of aggregation, but also to the higher degrees of freedom conferred by the peripheral residues.

The composition of the bacterial wall is the basis for bacterial classification and it is also responsible for different susceptibility to aPDT treatments [21]. Incorporation of intrinsic positive charges in the PS has been a benchmark in the reemerging of aPDT as a potential platform to fight antibiotic-resistant microbes [38,39,40]. Several studies have focused on establishing the relationships between PDI efficiency and the overall charge of the PS [25,41,42]. For example, Caminos *et al.* demonstrated that tricationic meso-substituted porphyrins outperformed, in terms of photosensitising efficiency, its mono-, di-, and tetracationic analogues against *E. coli* [41]. Despite **2** being endowed with a slightly lower Φ_Δ_ value both in water and MeOH, the substituent change does not modify its net charge as compared to **1**, which should result in a similar photoinactivation behavior. Porphycene **2** could successfully photoinactivate representative Gram-positive and Gram-negative bacterial cultures. In the case of *Candida* spp., the new tricationic compound showed worse efficiency than those reported for porphycene **1** [27]. Direct comparison between both tricationic PSs was assayed against two members of the ESKAPE pathogens, namely MRSA and *P. aeruginosa*. Both porphycenes were capable of inactivating bacterial cultures with efficiencies in the range of a 6-log_10_ reduction in the optimal conditions, both in a light- and concentration-dependent manner. Only differences below 1-log_10_ could be noticed between both compounds. However, one remarkable difference was the noteworthy dark toxicity of **1** at the highest concentration needed to photoinactivate *P. aeruginosa*. Of note is the fact that **1** had previously been unable to inactivate *P. aeruginosa* even at 100 μM concentration and 100 J·cm^−2^ fluence [27]. This difference in *P. aeruginosa* photoerradication is known to depend strongly on the experimental conditions [43]. In our case, the main explanations to this discrepancy can be attributed to (i) the difference in light source and (ii) the differences between the *P. aeruginosa* strains tested in the two studies (ATCC 19660 *vs.* ATCC 25668). For instance, it has been described that the ATCC 19660 strain (former study) is especially virulent when inoculated in an area of burned skin, producing septicemia in mice.

Overall, photoinactivation results are similar for both compounds despite the different charged substituents. Moreover, the optimal incubation times, fluences, drug concentrations, and photokilling efficiency lie within the same order of magnitude as those reported for other recently reported PSs [43,44,45,46,47,48,49,50]. Table 2 collects the optimal conditions for the novel porphycene **2**, along with appropriate references on aPDT studies against microorganisms of the same type. However, direct assessment among compounds and studies is not possible since the conditions are far from comparable.

**Table 2:** Optimal conditions for photoinactivation of microbial pathogens with porphycene 2. References to related aPDT studies for comparison.

Microorganism	Concentration (μM)	Fluence (J·cm^−2^)	Log_10_ Reduction	Other Studies
*S. aureus*	2	20	6	[27,43,45]
MRSA	0.5	22.5	6	[27,44,45,46,48]
*E. coli*	10	60	6	[27,43,45,48]
*P. aeruginosa*	20	100	6	[27,43,45,47,48]
*C. albicans*	35	30	>4	[27,49,50]
*C. krusei*	10	30	>4	[27,49,50]

Comparison of the photoinactivation results with and without removing the excess PS from the solution reveals a minor difference of approximately 1-log_10_ CFU reduction for MRSA, while no differences can be appreciated for *P. aeruginosa.* This finding indicates that both PSs bind strongly to the bacterial cells, as already observed for porphycene **1** [27]. Moreover the fact that PS molecules located in the bulk external aqueous phase are of little relevance for photoinactivation is consistent with the observation that their Φ_Δ_ value is two orders of magnitude lower in water than in less-polar environments such as methanol (Table 1) or the cell wall.

Flow cytometry analysis in combination with fluorescence techniques has proven useful for the assessment of cell functions including reproductive ability, metabolic activity, membrane integrity, and membrane potential [51]. It has also been applied to study the effects of different stress conditions on the viability of different microorganisms [52,53,54] and to investigate the antibacterial activity of essential oils [55,56]. In this work we have taken advantage of the solvent-dependent fluorescence properties of the porphycene to further support our previous hypothesis. Provided that two orders of magnitude also separate the Φ_F_ value in water and MeOH, we can make use of this property to keep track of the amount of PS that remains attached to the bacterial membrane (disaggregated and, thus, fluorescent) before and after the washing step to remove the excess PS. Our flow cytometry data, presented in Figure 6 and Table S1, are consistent with the observed minor effect of washing on the photoinactivation results. Specifically, the measured fluorescence intensity does not change significantly (*p* = 0.33) after removing the PS excess, confirming strong binding of the PSs to the cells. Moreover, in the case of porphycene **2** applied to *P. aeruginosa,* two different populations could be observed, particularly before removing the PS excess. This seems to indicate a non-homogenous interaction of this particular PS with the complex cell wall of *P. aeruginosa*, which could be important for the photoinactivation outcome. This observation points out the value of flow cytometry to examine cell binding.

Overall, we infer three ideas to account for the similar results between porphycenes **1** and **2**: (i) the changes in the peripheral tertiary amine substituent do not provide a big difference in the hydrophobicity-hydrophilicity balance given the high hydrophobicity of the macrocycle; (ii) both compounds have the same number and charge distribution, which is likely to cause similar effect when interacting with bacteria; and (iii) finally, but not least important, strong binding to the bacterial membrane (organic-like environment) renders both porphycenes disaggregated and, thus, highly active.

## 3. Experimental Section

### 3.1. Chemicals

Phosphate buffer saline (PBS) solutions were prepared by dissolving the required amount of a PBS tablet (Sigma –Aldrich, St. Louis, MO, USA) in 100 mL milliQ water. All other reagents were purchased also from Sigma-Aldrich (St. Louis, MO, USA) and were used as received. MeOH used for spectroscopic measurements was UV-grade.

### 3.2. Synthesis

2,7,12-Tris(α-pyridinio-*p*-tolyl)-17-(*p*-(methoxymethyl)phenyl) porphycene (Py_3_MeO-TBPo, **1**) was synthesized as previously described [27]. The 2,7,12-tris(trimethylamonioum-*p*-tolyl)-17-(*p*-(methoxymethyl)phenyl) porphycene analog (NMe_3_MeO-TBPo, **2**) was achieved by replacing the tribrominated porphycene derivative with trimethylammonium cations instead of pyridinium cations by using excess trimethylamine in inert solvent (see Scheme S1 and Figure S3 in the Supplementary Materials). Purity of **2** was confirmed by thin layer chromatography (TLC) using a pre-coated TLC plate (Silica gel C18 0.25 mm; Macherey-Nagel, Düren, Germany) in a trifluoroacetic acid/acetonitrile mixture (20:80), providing a unique spot at *R*_f_ = 0.26. ^1^H-NMR (δ/ppm, d6-MeOD): 10.02 (brs, 4H), 9.98 (brs, 4H) 8.58 (d, 6H), 8.38 (d, 2H), 7.84 (d, 2H), 8.06 (d, 6H); 4.86 (s, 6H), 4.58 (s, 2H); 3.72 (s, 2H), 3.59 (s, 2H), 3.30 (s, 27H). ^13^C NMR (δ/ppm, d6-MeOD): 160.3, 146.5, 141.6, 133.4, 133.3, 131.8, 131.7, 131.1, 129.9, 128.3, 128.2, 127.2, 124.2, 69.2, 69.1, 61.2, 59.0, 52.0, 14.1, 13.4.

UV/Vis (MeOH): λ (ε/M^−1^·cm^−1^) = 657 (46,483), 626 (41,129), 584 (32,590), 393(80,599), 377 (102,543) nm.

HRMS (ESI-TOF) *m*/*z* C_58_H_64_N_7_O_1_^3+^ calcd, 291.5052; found, 291.5054. Fragments for [M-3Br]^3+^ and [M-2Br]^2+^ confirm Br^−^ as the counter-ion.

### 3.3. Spectroscopic Techniques and Measurements

Absorption spectra were recorded with a Varian Cary 6000i spectrophotometer (Varian, Palo Alto, CA, USA). Fluorescence and excitation spectra were measured with a Fluoromax-4 spectrofluorometer (Horiba Jobin-Ybon, Edison, NJ, USA).

Time-resolved fluorescence experiments were carried out using a customized PicoQuant Fluotime 200 fluorescence lifetime system (PicoQuant GmbH, Berlin, Germany). Excitation was achieved by means of a 375 nm picosecond diode laser working at 10 MHz repetition rate and was observed at the emission maxima keeping the counting frequency below 1%. Fluorescence decays were analyzed using the PicoQuant FluoFit v4.6.5 data analysis software. The fluorescence quantum yield, Φ_F_, was determined by means of Equation (1):

(1) $${\text{Φ}}_{F,\;sample}=\frac{AUC_{sample}\;\cdot \;n_{sample}^{2}}{AUC_{reference}\;\cdot \;n_{reference}^{2}}\times {\text{Φ}}_{F,\;reference}$$

where *AUC*_i_ is the fluorescence intensity integrated over the entire emission spectrum corrected by the absorption factor (1 − 10^−Abs^) and *n*_i_ is the refractive index of the solvent used in each case. Cresyl violet in MeOH was used as reference (Φ_F_ = 0.54) [57].

For time-resolved phosphorescence detection, a diode-pumped Nd:YAG laser (FTSS355-Q, Crystal Laser, Berlin, Germany) was used for excitation at 532 nm working at 1 kHz repetition rate (1.2 μJ per pulse, 1 ns pulse-width). A 1064 nm rugate notch filter (Edmund Optics, York, UK) was placed at the exit port of the laser to remove any residual component of its fundamental emission in the NIR region. The luminescence exiting from the side of the sample was filtered by one long-pass filter of 1000 nm and narrow bandpass filters at either 1270 or 1110 nm to remove any scattered laser radiation and isolate the NIR emission. A TE-cooled Hamamatsu NIR sensitive photomultiplier tube assembly (H9170-45, Hamamatsu, Hamamatsu City, Japan) was used as detector. Photon counting was achieved with a multichannel scaler (PicoQuant’s Nanoharp 250). Time-resolved ^1^O_2_ phosphorescence signals (*S*_t_) are defined by three parameters, namely *S*_0_, τ_T_, and τ_Δ_ (Equation (2)).

(2) $$S_{t}=S_{0}\times \frac{\tau_{\text{Δ}}}{\tau_{\text{Δ}}-\tau_{T}}\times \left(e^{-t/\tau_{\text{Δ}}}-e^{-t/\tau_{T}}\right)$$

Actual *S*_t_ signals were analyzed using the PicoQuant data analysis software to extract lifetime (τ_T_ and τ_Δ_) and amplitude (*S*_0_) values. Quantum yields for ^1^O_2_ production (Φ_Δ_) were calculated from the amplitudes using the Equations (3) and (4).

(3) $$S_{0}\propto {\text{Φ}}_{\text{Δ}}$$

(4) $${\text{Φ}}_{\text{Δ},sample}={\text{Φ}}_{\text{Δ},ref}\times \frac{S_{0,sample}}{S_{0,reference}}$$

### 3.4. Flow Cytometry

The quantification of the binding of PSs was evaluated by flow cytometry. For these experiments, bacteria were subjected to the same treatments used for PDI experiments, but instead of being illuminated after incubation and washed they were analyzed with a FACSCanto^Tm^ II flow cytometer (Becton Dickinson, BD, San Jose, CA, USA). Samples were excited with the 488 nm laser, and fluorescence emission signals were recorded at wavelengths higher than 670 nm. The bacteria population was isolated from instrument noise by setting electronic gates on the dual-parameters dot plots of forward scatter against side scatter. For each sample, 20,000 events were acquired and analyzed with the FACSDiva software (Becton Dickinson, BD, San Jose, CA, USA). Samples not incubated with the PSs were used to determine the cell background fluorescence.

### 3.5. Microbial Techniques

#### 3.5.1. Microbial Strains and Growth Conditions

*Escherichia coli* CECT 101 and *Staphylococcus aureus* CECT 239 were obtained from the Spanish Type Culture Collection (CECT, Valencia, Spain). A methicillin-resistant strain of *Staphylococcus aureus* ATCC BAA-44, *Pseudomonas aeruginosa* ATCC 25668, *Candida albicans* ATCC 10231, and *Candida krusei* ATCC 6258 were purchased from LGC Promochem (Teddington, UK).

Bacterial cells were grown overnight in sterile tryptic soy broth at 37 °C. Stock inoculum suspensions were prepared in PBS and adjusted to an optical density at 600 nm of 0.4 for *E. coli* and 0.6 for *Pseudomonas aeruginosa* (equivalent to approx. 10^8^ colony-forming units).

Bacterial cells were aerobically grown overnight at 37 °C in brain-heart infusion (BHI), lysogeny broth (LB), or tryptic soy broth (TSB) to stationary phase. A reinoculum was then grown in fresh LB medium at 37 °C to an optical density at 600 nm (OD_600_) of 0.2 (start of log phase).

*Candida* spp. were grown overnight at 35 °C in Sabouraud broth, and then subcultured in fresh Sabouraud medium at 35 °C in an orbital shaking incubator at 130 rpm to an OD_600_ = 0.7, corresponding to approx. 10^7^ CFU/mL. The suspensions were then centrifuged (5 min, 3500 rpm) and re-suspended with sterile PBS at pH 7.4 at the same concentration for phototoxicity experiments.

Cultures were maintained for two weeks subcultured on tryptic soy agar, in the case of bacteria, or on Sabouraud agar medium for *Candida* strains.

#### 3.5.2. Photodynamic Inactivation Procedure

Cell suspensions in PBS were incubated in the dark at room temperature for 30 min with the appropriated amount of PS. Centrifugation (3 min, 12,000 rpm) of aliquots was used to remove the excess of PS that was not taken up by the bacteria when experiments required it. Then, bacterial suspensions aliquots were placed in 96-well plates. The wells were illuminated from the top of the plates. At the time points when the desired fluences had been delivered, aliquots were thoroughly mixed before sampling to avoid bacteria settlement. Light-alone controls (without PS) were also performed for all experimental conditions in order to rule out any inactivation due to the light and heating effects. For determination of population reduction, aliquots were serially diluted, streaked on nutrient agar plates, and incubated in the dark either at 37 °C for bacterial cells and *C. krusei*, or at 30 °C (*C. albicans*).

*E. coli*, *S. aureus*, and *Candida* species were illuminated from the top by a LED-based lamp emitting red light (Sorisa Photocare; 635 ± 15 nm). MRSA and *P. aeruginosa* cell suspensions were irradiated with a Waldmann PDT 1200 lamp (Waldmann Medizintechnik GmbH, Villingen-Schwenningen, Germany; 600–750 nm). Fluence rates were routinely measured using a power meter.

### 3.6. Statistical Analyses

Experiments were carried out in triplicate for each condition and the results are the mean of the three experiments. Differences between means were evaluated by the unpaired Student’s *t* test. *p* values of <0.05 were considered significant.

## 4. Conclusions

Two tricationic PSs have been assayed against bacterial and yeast cultures in order to assess their photochemical behavior in solution and their photokilling efficiency. Optical spectroscopy techniques in combination with flow cytometry have provided valuable insight into the photoinactivation process. Both compounds show a similar behavior in solution and are equally effective against the microorganisms tested, unlike other families of PSs. As is usually the case, higher concentrations of the porphycenes are needed to kill Gram-negative bacteria as compared to Gram-positive ones. Both PSs are largely aggregated in aqueous environments, losing their photosensitization ability. As a consequence, PS molecules located in the bulk external aqueous phase do not contribute significantly to the photoinactivation of microorganisms. However, flow cytometry studies reveal a high affinity of both porphycenes for both Gram-positive and Gram-negative bacterial cells, which results in tight binding. Bound PSs recover their photodynamic activity. Our findings further reinforce the potential of cationic porphycenes as putative therapeutic agents for aPDT.

## Supplementary Materials

Supplementary materials can be found at http://www.mdpi.com/1422-0067/16/11/25999/s1.
