# Supplementary material for: A Comparative Study on Two Cationic Porphycenes: Photophysical and Antimicrobial Photoinactivation Evaluation
Source: Int J Mol Sci. 2015 Nov 12;16(11):27072–86. doi: 10.3390/ijms161125999 (PMC4661857; doi:10.3390/ijms161125999)
Supplement: Supplementary file 1 [file ijms-16-25999-s001.pdf]

Supplementary Materials: A Comparative Study on Two Cationic Porphycenes: Photophysical and Antimicrobial Photoinactivation Evaluation

Rubén Ruiz-González, Montserrat Agut, Elena Reddi and Santi Nonell

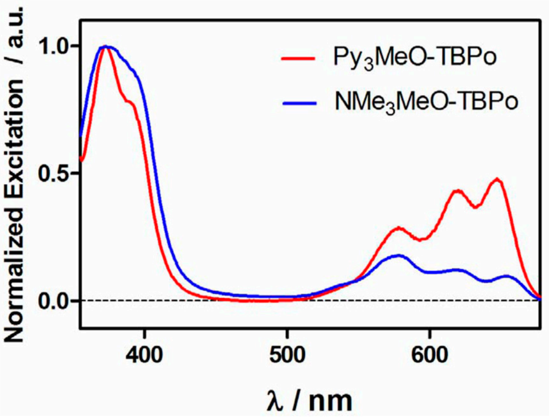

Figure S1. Excitation spectra of the porphycene's fluorescence at 700 nm in water.

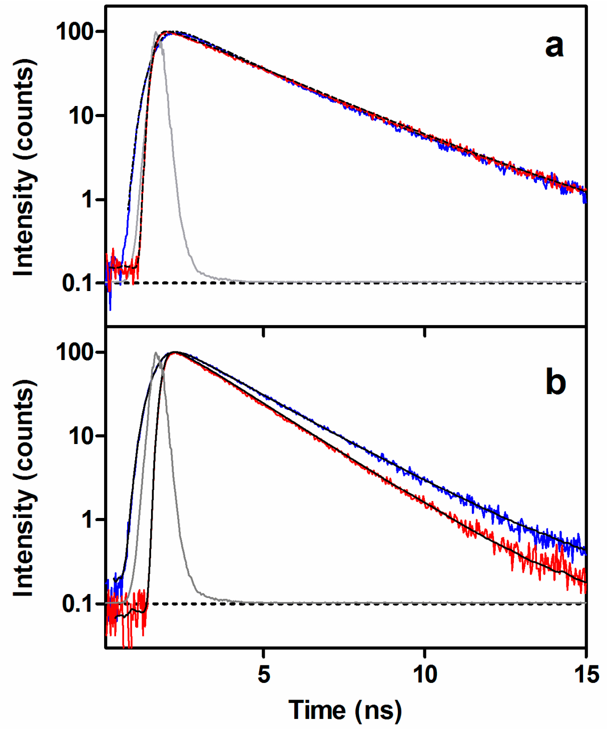

Figure S2. Time-resolved fluorescence decays in MeOH (**a**) and water (**b**) for porphycene **1** (red) and **2** (blue). Signal, fit (black) and instrument's response function (grey) at 700 nm upon excitation at 375 nm.

Table S1. Flow cytometry fluorescence distribution upon binding experiments.

Compound	Fluorescence Intensity/Counts		
	PS Removal (Washing)	MRSA	*P. aeruginosa*
**Py3MeO-TBPo**	NO	2600 ± 900	4200 ± 1000
	YES	2100 ± 400	4300 ± 2000
**NMe3MeO-TBPo**	NO	2600 ± 700	2600 ± 900
	YES	2000 ± 900	2600 ± 700

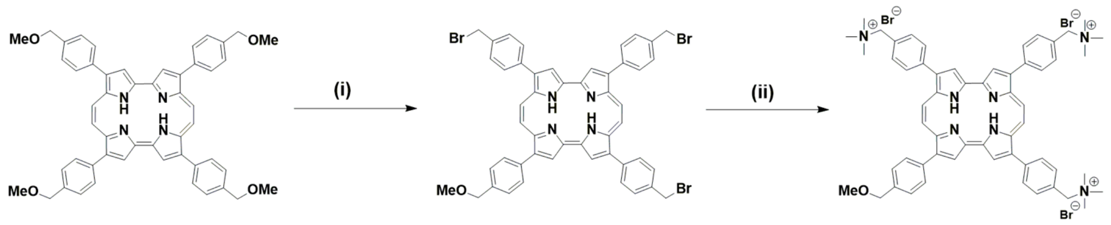

Scheme S1. Synthetic route to compound **2**. (i) HBr; (ii) NMe_3.

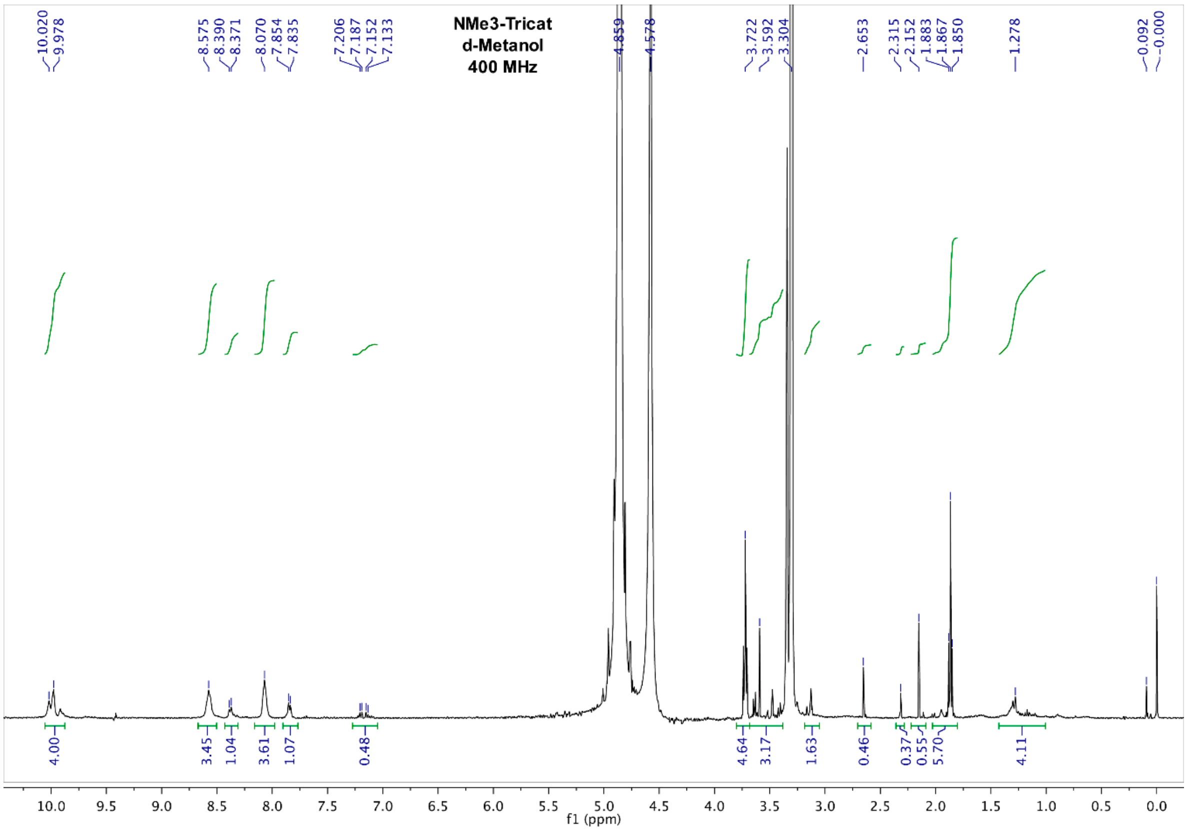

Figure S3. ^{1}H-NMR of porphycene **2** in deuterated methanol.
